# Genomic characterization provides new insight into *Salmonella* phage diversity

**DOI:** 10.1186/1471-2164-14-481

**Published:** 2013-07-17

**Authors:** Andrea I Moreno Switt, Renato H Orsi, Henk C den Bakker, Kitiya Vongkamjan, Craig Altier, Martin Wiedmann

**Affiliations:** 1 Department of Food Science, Cornell University, Ithaca 14853, New York; 2 Department of Population Medicine and Diagnostic Sciences, College of Veterinary Medicine, Cornell University, Ithaca, New York

**Keywords:** *Salmonella* phage, Phage genomics

## Abstract

**Background:**

*Salmonella* is a widely distributed foodborne pathogen that causes tens of millions of salmonellosis cases globally every year. While the genomic diversity of *Salmonella* is increasingly well studied, our knowledge of *Salmonella* phage genomic diversity is still rather limited, despite the contributions of both lysogenic and lytic phages to *Salmonella* virulence, diversity and ecology (e.g., through horizontal gene transfer and *Salmonella* lysis). To gain a better understanding of phage diversity in a specific ecological niche, we sequenced 22 *Salmonella* phages isolated from a number of dairy farms from New York State (United States) and analyzed them using a comparative genomics approach.

**Results:**

Classification of the 22 phages according to the presence/absence of orthologous genes allowed for classification into 8 well supported clusters. In addition to two phage clusters that represent novel virulent *Salmonella* phages, we also identified four phage clusters that each contained previously characterized phages from multiple continents. Our analyses also identified two clusters of phages that carry putative virulence (e.g., adhesins) and antimicrobial resistance (tellurite and bicyclomycin) genes as well as virulent and temperate transducing phages. Insights into phage evolution from our analyses include (i) identification of DNA metabolism genes that may facilitate nucleotide synthesis in phages with a G+C % distinct from *Salmonella*, and (ii) evidence of *Salmonella* phage tailspike and fiber diversity due to both single nucleotide polymorphisms and major re-arrangements, which may affect the host specificity of *Salmonella* phages.

**Conclusions:**

Genomics-based characterization of 22 *Salmonella* phages isolated from dairy farms allowed for identification of a number of novel *Salmonella* phages. While the comparative genomics analyses of these phages provide a number of new insights in the evolution and diversity of *Salmonella* phages, they only represent a first glimpse into the diversity of *Salmonella* phages that is likely to be discovered when phages from different environments are characterized.

## Background

*Salmonella* is an important and globally distributed foodborne pathogen, which causes an estimated 93 million gastroenteritis cases, and 150,000 deaths annually, among the global human population [1]. In the United States, *Salmonella* causes an estimated 1 million annual human cases and is the leading reported cause of death and hospitalization related to foodborne disease [2]. This pathogen is principally acquired by the consumption of contaminated food, although contact with infected animals and human to human transmission are also known transmission routes of *Salmonella*[3]. Dairy cattle and dairy products are important sources of *Salmonella*; a number of serovars ranked in the top 10 *Salmonella* serovars among human cases in the U.S. (e.g., Newport, Typhimurium) are commonly isolated from dairy cattle [4-6]. Whereas several studies have reported the prevalence and distribution of *Salmonella* on dairy farms, there is limited data on *Salmonella* phage distribution in this environment. Recently, our group reported a high prevalence and diversity of *Salmonella* phages on dairy farms in rural areas of New York State, and also identified closely related phages on farms hundreds of miles apart [7]. We thus elected to use phage isolates from dairy farm environments as a model to explore the genomic diversity of *Salmonella* phages associated with a specific environment.

Bacteriophages are the most abundant biological entities on this planet, show a high degree of host specificity, and phages are present in all the environments where a suitable host is found [8]. Phage populations have been estimated to be highly dynamic, for example, it has been estimated that approx. 10^23^ phage infections per second occur globally in marine environments [9]. Consequently, phages play pivotal roles in bacterial evolution, from killing bacteria to being agents of horizontal gene transfer [8,10,11]. Several *Salmonella* phages and prophages have been reported (e.g., Fels-1, Gifsy-2, P22, FelixO1), and selected phages have been fully sequenced [8,12-16]. Currently, there are genome sequences available for *Salmonella* phages from different regions of the world (e.g., U.S., U.K., Canada, and South Korea), isolated from diverse animal production facilities (e.g., swine and poultry), and with different host specificity (e.g., able to lyse either *Salmonella* serovars Typhi, Typhimurium, or Enteritidis). Regardless of the previously available phage genome sequences, the diversity of *Salmonella* phages is still severely under-sampled and our knowledge of the genomic diversity of *Salmonella* phages associated with different environments is very limited.

## Results and discussion

The fact that *Salmonella* is well recognized as a diverse species, with >2,600 serovars, various patterns of transmission, and host specificity, has led to considerable recent efforts [17-19] to characterize the *Salmonella* pangenome and to probe the diversity of this pathogen at the genomic level. By comparison, our knowledge of *Salmonella* phage genomic diversity has remained rather limited; in big part due to under-sampling. We thus selected 22 *Salmonella* phages, with a range of different phenotypic and genotypic characteristics [7], from nine dairy farms for genome sequencing to characterize the *Salmonella* phage diversity in environments (dairy farm) and hosts (cattle) where *Salmonella* is commonly found. Major findings of this study include (i) identification, by cluster analysis, of two *Salmonella* phage orthoclusters that have not been previously reported, (ii) identification of four phage clusters containing phages previously identified in various locations worldwide, (iii) identification of phages carrying putative antimicrobial resistance and virulence genes, (iv) identification of DNA metabolism genes in *Salmonella* phages with G+C contents different from *Salmonella*, and (v) evidence of *Salmonella* phage tailspike and fiber diversity due to both single nucleotide polymorphisms and major re-arrangements.

### Identification, by cluster analysis, of two *Salmonella* phage orthoclusters that have not been previously reported

**Figure 1:**
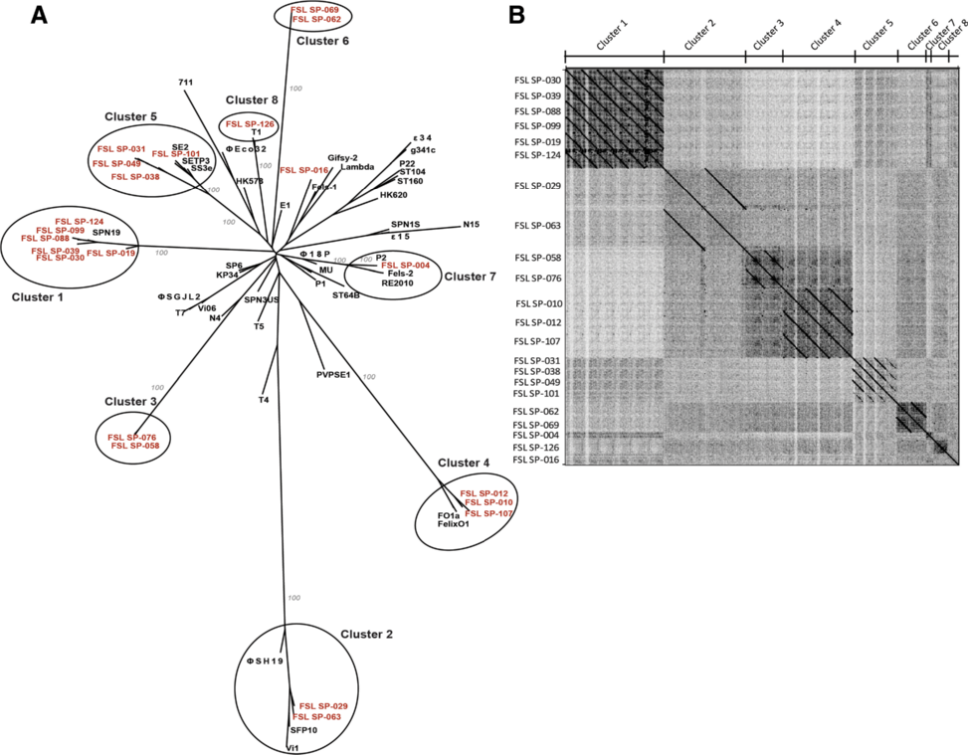
**Genomic comparisons of the phages sequenced here. A)** A neighbor joining tree based on presence/absence of orthologous gene families. This tree includes the phages sequenced here (in red) as well as previously sequenced *Salmonella* phages and known phage genera infecting *Enterobacteriaceae* (in black; see Additional file 1). Circles indicate the 8 phage clusters that contained phages characterized here; bootstrap values (grey numbers, based on 1,000 replicates) are shown for selected branches that lead to these 8 clusters. **B)** Dot plot comparison of nucleotide identity of the 22 phages sequenced here. This comparison was performed with Gepard [79]. The vertical axis shows the phage IDs and the horizontal axis indicates the phage clusters; the apparent diagonal lines indicate high levels of nucleotide identity; while each phage shows 100% identity to itself (displayed as a diagonal line), comparisons of two phages that differ only by a few SNPs (e.g., cluster 6 phages FSL SP-062 and FSL SP-069, which differ by 4 SNPs) will also show up as an apparent uninterrupted line as a small number of differences is not visible with the resolution possible.

The 22 newly sequenced phage genomes ranged in size from 30 to 158 kb with G+C contents ranging from 39 to 56% (Table 1); previously sequenced *Salmonella* phages ranged in genome size from 33 to 240 kb and showed G+C contents between 39 to 53% [12,14,20,21] (see Additional file 1 for details). Annotation of the genomes sequenced here identified between 45 and 264 genes in a given phage, which is consistent with reports on previously sequenced *Salmonella* phages [8,20]. A total of 8 of the 22 phages sequenced here contained known lysogeny modules, suggesting a temperate life cycle (Table 1). To initially characterize the diversity of these phages, we clustered phage genomes based on the presence/absence of families of orthologous genes, similar to a method previously used to cluster Mycobacteriophage genomes [22-24]. We refer to this approach as an orthoclustering analysis, and will refer to the resulting clusters of phages as orthoclusters. A total of 65 phage genomes were included in this analysis, including the genomes for (i) 22 phages characterized here, (ii) 23 *Salmonella* phages that have previously been reported, and (iii) 20 phages that represent type species of known phage genera infecting *Enterobacteriaceae*[25] (Additional file 1). This analysis revealed eight distinct and well supported (100% bootstrap support) clusters of phage genomes; 21 of the 22 new phage genomes reported here grouped into one of these eight well supported clusters (Figure 1A). Overall, the eight clusters identified here contained phages with high levels of overall genome homology. For example, the seven phages in cluster 1 shared 62/73 orthologous genes with amino acid (aa) identities for these genes ranging from 72 to 100% (93% average aa identity across all orthologous genes). The five phages in cluster 4 shared 98/133 orthologous genes with aa identities for these genes ranging from 68 to 100% (91% average aa identity across all orthologous genes). The genome wide nucleotide identity of phages sequenced here was also compared using a dot plot (Figure 1B), which supported the cluster relationships that were derived based on the orthocluster analysis. For example, phage FSL SP-101 falls in a separate branch from the other FSL phages in cluster 5 (FSL SP-031, FSL SP-038 and FSL SP-049; Figure 1A), and the dot plot analysis shows that FSL SP-101 displays low sequence similarity (as demonstrated by the interrupted line on the diagonal in the comparisons) when compared to the other phages in cluster 5 (Figure 1B), This result is in agreement with the considerable nucleotide divergence observed between FSL SP-101 and the other FSL phages in this cluster (e.g., FSL SP-101 and FSL SP-031 showed only 53.8% nucleotide identity over the whole genome).

**Table 1:** Characteristics of newly sequenced phages

**Phage**	**Farm**	***Salmonella*serovar host**	**Genome size (kb)**	**GC %**	**Phage cluster**^**2**^	**Putative family**^**3**^	**Putative life cycle**^**5**^	**Related *Salmonella *phage**	**GenBank accession no.**
FSL SP-030	8	Dublin	59	56.6	1	*Siphoviridae*	temperate	SPN19	KC139519
FSL SP-039	8	Cerro	59	56.6	1	*Siphoviridae*	temperate	SPN19	KC139514
FSL SP-088	10	Typhimurium	59	56.4	1	*Siphoviridae*	temperate	SPN19	KC139512
FSL SP-099	13	Newport	59	56.6	1	*Siphoviridae*	temperate	SPN19	KC139667- KC139680
FSL SP-019	3	Newport	59	56.4	1	*Siphoviridae*	temperate	SPN19	KC139571- KC139631
FSL SP-124	15	Cerro	59	56.5	1	*Siphoviridae*	temperate	SPN19	KC139515
FSL SP-029	8	Dublin	158	45.0	2	*Myoviridae*	virulent	V01, SFP10	KC139560- KC139570
FSL SP-063	9	Dublin	156	44.9	2	*Myoviridae*	virulent	V01, SFP10	KC139522- KC139525
FSL SP-058	3	Dublin	72	39.6	3	*Podoviridae*	virulent	none	KC139517
FSL SP-076	9	Dublin	72	39.5	3	*Podoviridae*^4^	virulent	none	KC139520
FSL SP-010	2	Mbandaka	87	39.4	4	*Myoviridae*	virulent	Felix O1	KC139526- KC139542
FSL SP-012	1	Mbandaka	87	39.3	4	*Myoviridae*	virulent	Felix O1	KC139543- KC139556
FSL SP-107	13	Mbandaka	88	39.3	4	*Myoviridae*	virulent	Felix O1	KC139638- KC139648
FSL SP-031	8	Cerro	44	51.3	5	*Siphoviridae*	virulent	SE2, SS3e	KC139518
FSL SP-038	6	Cerro	42	51.1	5	*Siphoviridae*	virulent	SE2, SS3e	KC139652- KC139666
FSL SP-049	6	Cerro	43	50.9	5	*Siphoviridae*	virulent	SE2, SS3e	KC139557- KC139559
FSL SP-101	13	Dublin	41	50.2	5	*Siphoviridae*	virulent	SE2, SS3e	KC139511
FSL SP-062^1^	9	Newport	56	42.8	6	*Siphoviridae*^4^	virulent	none	KC139632- KC139637
FSL SP-069^1^	9	Newport	56	42.8	6	*Siphoviridae*	virulent	none	KC139649- KC139651
FSL SP-004	1	Newport	30	52.8	7	*Myoviridae*^4^	temperate	P2, PSP3	KC139521
FSL SP-126	15	Kentucky	51	42.9	8	*Siphoviridae*	virulent	T1	KC139513
FSL SP-016	2	Anatum	46	50.2	-	*Siphoviridae*	temperate	Gifsy-2, Fels-1	KC139516

^1^ FSL SP-062 and FSL SP-069 only differ in 4 single nucleotide polymorphisms; therefore, FSL SP-069 is a variant of FSL SP-062.

^2^ phage orthoclusters were determined based on presence/absence of orthologous genes.

^3^ putative families were determined *in silico* as described in Methods.

^4^ families were also confirmed by transmission electron microscopy.

^5^ presence of lysogeny module was used to identify the putative life cycle of a given phage isolate.

Phage FSL SP-016, a 46 kb phage, which contains a known lysogeny module (Table 1; Additional file 2), could not be assigned a specific cluster (Figure 1A). This phage clustered with the *Salmonella* phage Gifsy-2 and Fels-1 (although not supported by a significant bootstrap value [<50%]) and shares 18/60 ORFs with Gifsy-2 [GenBank: NC_010393], with an average aa sequence identity of 87%. In addition, two of the new phage genomes reported here (FSL SP-004 and FSL SP-126) grouped into clusters that only contained other phages isolated from the U.S. FSL SP-004 has a 30 kb genome that contains a known lysogeny module (Table 1, Additional file 2), indicating that this is a temperate phage. This phage groups into cluster 7, along with P2, the type species of the genus *P2likevirus*. This genus also includes the Enterobacteria phage PSP3 [GenBank: NC_005340], which also infects *Salmonella*[26]; FSL SP-004 and PSP3 present an average aa identity of 92% among the 22 ORFs shared. FSL SP-126, a 51 kb phage (Table 1) that appears to be virulent (as no known lysogeny module was identified in the genome), clustered with Enterobacteria phage T1 [GenBank: NC_005833], which is the type species of *Tunalikevirus.* Among the 83 ORFs in FSL SP-126, 37 have an ortholog in T1 (the average aa identity for these 37 orthologs is approx. 90%).

Five of the eight phage clusters identified here contain phages that were isolated from different farms (see Table 1). For example, cluster 1 contains 6 phages isolated from five farms (farms 3, 8, 10, 13, and 15), while cluster 4 contains 3 phages isolated from three farms (farms 1, 2, and 13). This finding is consistent with previous pulsed field gel electrophoresis (PFGE) and restriction fragment length polymorphism (RFLP) data [7], on these as well as additional phages, which also found evidence for isolation of similar phages on dairy farms hundreds of miles apart. Combined, these data suggest that these phages may either be genetically stable and endemic or easily dispersed among farms (e.g., through animal movement, traffic between farms, fomites that move between farms, etc.), consistent with the fact that *Salmonella* is commonly found on dairy farms [4,27] and can be transmitted between farms through various means [28,29].

**Figure 2:**
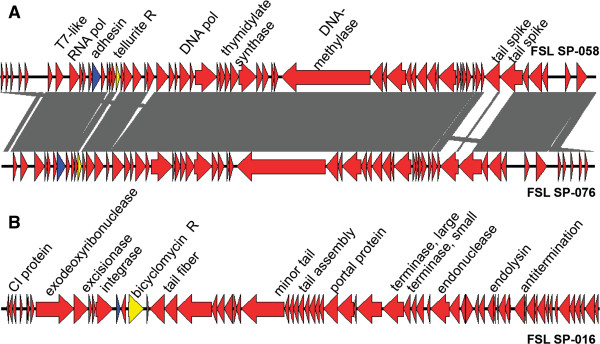
**Linear representation of phages carrying antimicrobial resistance and virulence genes. (A)** Comparison, using the BLAST algorithm, of phages in cluster 3 (FSL SP-058 and FSL SP-076), grey shaded regions are regions of homology between these two phages. **(B)** Linear representation of the temperate phage FSL SP-016. Red arrows indicate ORFs, blue arrows indicate putative virulence genes, and yellow arrows indicate putative antimicrobial resistance genes.

Two of the clusters identified here (clusters 3 and 6) only contained the genomes of *Salmonella* phages isolated here; these two clusters thus represent groups of *Salmonella* phages that have not been previously reported and their genomic characteristics are briefly described here. Cluster 3 includes two phages (FSL SP-058 and FSL SP-076; Table 1) that were obtained from 2 different farms. Ninety-seven ORFs were found in the phages of this cluster, functionally annotated ORFs include, for example, DNA polymerase, RNA polymerase, tailspikes, and terminase subunits (Additional file 2); aa identity among 88 orthologs found in both phages ranged from 65 to 100% (89% average aa identity). While these two phages do not contain a lysogeny module, suggesting that they are virulent, further phenotypic characterization of these phages is needed to confirm a virulent life cycle. Phages FSL SP-058 and FSL SP-076 share a conserved backbone, but show considerable variation in the two tailspike encoding genes (Figure 2A), which, as discussed below in detail, is often related with host range variability [30]. A BLASTP search of all cluster 3 ORFs against GenBank revealed no matching phages, suggesting that phages in cluster 3 appear to be novel virulent *Salmonella* phages.

**Figure 3:**
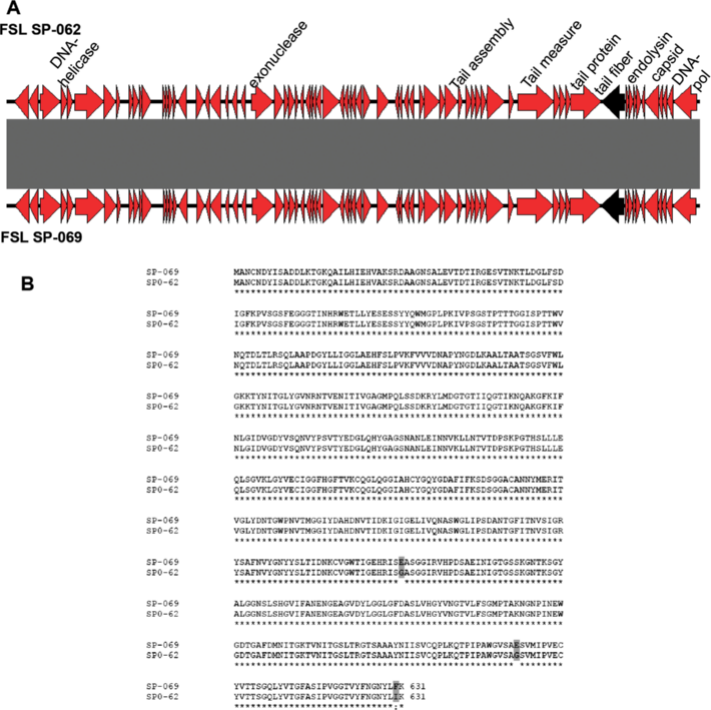
**Linear representation of novel phages of cluster 6. (A)** Comparison of FSL SP-062 and FSL SP-069 using the BLAST algorithm, red arrows indicate ORFs, black arrows indicate the tail fiber, and grey shaded regions are regions of homology between these two phages. **(B)** Alignment of the tail fibers amino acid sequences, the three shaded amino acids indicate substitutions.

Cluster 6 is the second cluster that includes only phages characterized here; the two phage isolates in this cluster (FSL SP-062 and FSL SP-069; Table 1) were obtained from the same farm. We identified 102 ORFs in the phages of this cluster, 85 of them are hypothetical proteins and only 17 ORFs could be functionally annotated (Additional file 2 and Figure 3A). A lysogeny module is also absent in these two phages. Interestingly, only four single nucleotide polymorphisms (SNPs) differentiate phage isolates FSL SP-062 and FSL SP-069, with three of these SNPs located in the gene encoding the tail fiber; these two phage isolates thus should be considered variants rather than different phages (Table 1). Similar to cluster 3, a BLASTP search of all cluster 6 ORFs against GenBank revealed no matching phages, suggesting that the cluster 6 phages also represent novel *Salmonella* phages. Overall, we thus identified a number of novel *Salmonella* phages in this study, consistent with many reports that bacteriophages are extremely under-sampled and that with more environments and locations being sampled, more novel phages are likely to be identified [22,23,31].

### Identification of four phage clusters containing phages previously identified in various locations worldwide

In addition to two phage orthoclusters that only contained phages characterized here, we also identified four clusters (clusters 1, 2, 4, and 5) that each contains phages sequenced here as well as previously sequenced *Salmonella* phages obtained from other locations throughout the globe. Phage cluster 1 contains six very similar phages (isolated from 5 farms in the U.S.) as well as one phage that was isolated in South Korea (SPN19), which has not been assigned a genus to date [32]. These phages have a syntenic genome (Additional file 3), a size of approx. 59 kb, and a G+C content of 56.5% (Table 1). Annotations identified genes encoding phage morphogenesis and replication proteins (Additional file 2). A lysis control module was identified in the 6 phage genomes sequenced here, but not in phage SPN19. Interestingly, phages FSL SP-030 and FSL SP-039, which cluster together based on the overall homology from the MAUVE alignment, were found to carry a different lysogeny control module (a Cro/C1 protein [33]) as compared to the other four phages, which encode a lysogeny control module that shows homology to the phage related helix-turn-helix XRE-family of transcriptional regulators [34]. Further analyses showed that the genomes of phages in cluster 1 resemble the genome of *Enterobacter cancerogenes* phage Enc34 [35] [GenBank: JQ340774], a phage of the *Siphoviridae* family that has not been assigned a genus to date, according to the International Committee on Taxonomy of Viruses 2012 [25]; this phage was hence not included in the cluster analysis shown in Figure 1. A *Enterobacter* phage Enc34 shows synteny with cluster 1 phages. While there are 11 hypothetical proteins present in phage Enc34, but not in the cluster 1 phages, 40 orthologs were found in both Enc34 and the cluster 1 phage FSL SP-088 (Additional file 4); the average aa identity among these shared ORFs is approx. 75%. These data indicate that phages similar to cluster 1 have previously been isolated from *Enterobacteriaceae* hosts other than *Salmonella*.

**Figure 4:**
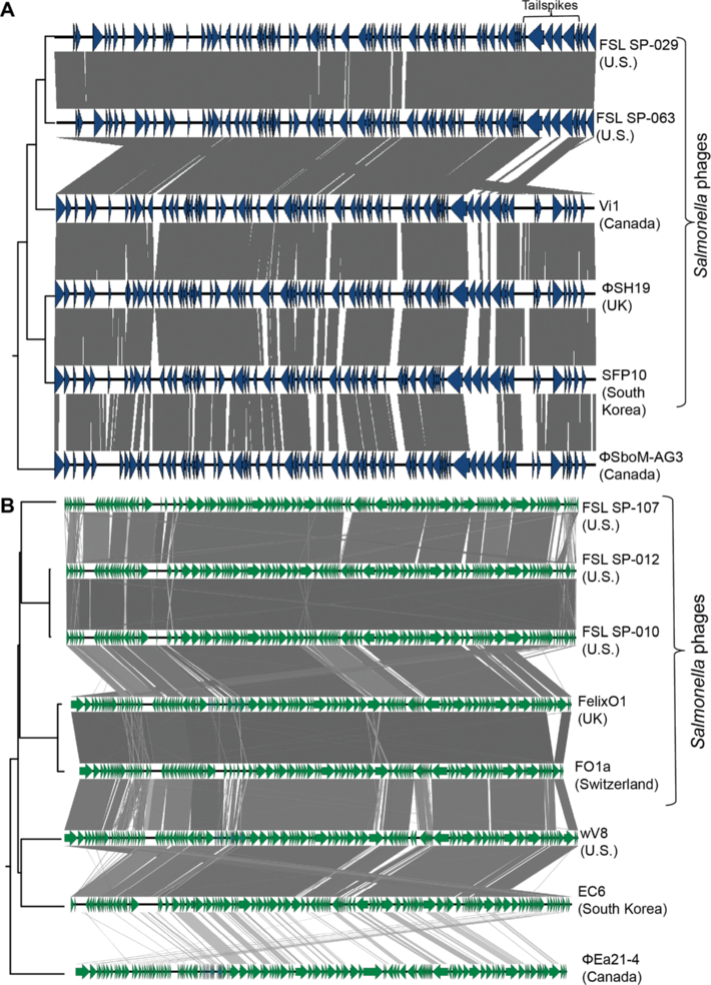
**Comparison, using the BLAST algorithm, of phages in clusters 2 and 4. (A)** Comparison of phages in cluster 2, representing two phages sequenced in this study (FSL SP-029 and FSL SP-063) and four phages previously reported in Canada, U.K., and South Korea, blue arrows indicate ORFs. **(B)** Comparison of phages in cluster 4, representing three phages sequenced in this study (FSL SP-010, FSL SP-012, and FSL SP-107) and five phages previously reported in Canada, U.S., South Korea, and U.K. Phages and country of origin are indicated on the right, green arrows indicate ORFs, and grey shaded regions are regions of homology. On the left side is the tree generated with the Mauve algorithm indicating the overall homology of the phages.

**Figure 5:**
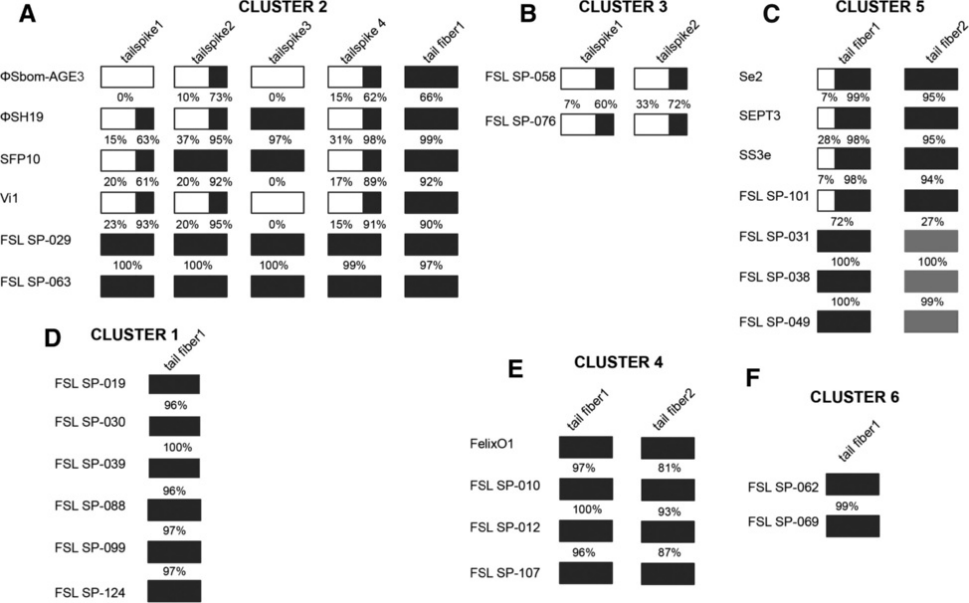
**Representation of variations in tailspikes and fibers in six phage clusters.** Tailspikes and fibers are represented as boxes, while white boxes represent low (< 37%) or no identity, black boxes represent an identity > 60%. In panels **A**, **B**, and **C** are the phage clusters that presented high diversity in their tailspikes and fibers. In panels **D**, **E**, and **F** are the phage clusters that presented low diversity in their tailspikes and fibers. The analyses consisted in pairwise comparisons of the amino acids identity. Identity percentage for tailspike or fiber is indicated for pairs of phages that are shown next to each other in this figure (see Additional file 6 for the complete pairwise comparison). Grey boxes in cluster 5 represent the allele of tail fibers only found in phages FSL SP-031, FSL SP-038, and FSL SP-049.

Cluster 2 contains five *Salmonella* phages (Figure 1A), two sequenced in this study (obtained from 2 different farms) as well as three previously reported *Salmonella* phages (i.e., Vi1, ΦSH19, SFP10, isolated from Canada, U.K., and South Korea, respectively) (Figure 1A). In addition, one previously reported *Shigella* phage (ΦSboM-AG3, isolated from Canada) showed homology with the phages in this cluster (Figure 4A) [36]; a recent publication proposed these phages as “*Viunalikevirus*” [37], a new phage genus in the family *Myoviridae*. Phages belonging to cluster 2 possess large (approx. 158 kb) genomes (Table 1); in the two phages sequenced here (i.e., FSL SP-029 and FSL SP-063), we identified 204 ORFs, 81 of them were functionally annotated (Additional file 2). While ORFs involved in phage morphogenesis, replication and DNA metabolism were found to be conserved among these six phages, genes involved in host specificity (i.e., tail fibers and tailspikes) showed considerable diversity (Figure 5), consistent with previous reports that these genes often show diversity due to recombination and therefore considerable mosaicism [12,36,38,39]. Despite their isolation from three continents, phages in cluster 2 present an overall genome synteny as well as high level conservation (aa identity of 65 to 100% across 115 homologs found in all 6 genomes; average aa identity of 89%) (see Figure 4A). Interestingly, this conservation appears to be stable in both time and space; *Salmonella* phage Vi1, which is the type species of this genus was isolated in Canada in the 1930’s [38].

Cluster 4 includes three *Salmonella* phages sequenced in this study (i.e., FSL SP-010, FSL SP-012, and FSL SP-107, each isolated from a different farm), one *Salmonella* phage isolated in Switzerland (FO1a [GenBank: JF461087]), and one well characterized *Salmonella* phage isolated in the U.K. in 1943 (i.e., FelixO1) [40]. The five phages in this cluster have an approximate genome size of 87 kb (Table 1); aa identity among 98 shared orthologs ranged from 68 to 100% (average aa identity of approx. 91%). FelixO1 is the type species of the genus *Felixounalikevirus*, this phage genus also includes *Salmonella* phage SPT-1, isolated in the U.S., *E. coli* phage wV8, isolated in the U.S., *E. coli* phage EC6, isolated in South Korea, and *Erwinia* phage ΦEa21-4, isolated in Canada [21,40,41] (Figure 4B). We thus not only identified three new members of the genus *Felixounalikevirus*, but also provided further evidence that *Felixounalikeviruses* are widely distributed. Whole genome alignments, showed that phage FO1a is the closest to FelixO1, followed by the three *Salmonella* phages sequenced in this study (Figure 4B); the most distinct phage genome among the *Felixounalikeviruses* is *Erwinia* phage ΦEa21-4, which only shares some ORFs with these phages, but lacks ORFs conserved among the rest of the phages in this genus (see Figure 4B). The major difference between FelixO1 and phages sequenced here were found to be insertions and deletions of homing endonucleases, which are mobile elements that have been reported in some phages [42]. Interestingly, genes encoding homing endonucleases are often inserted between genes or inserted into genes that appear conserved among these closely related phages. While high numbers of genes encoding homing endonucleases were reported for FelixO1 [40], we here provided evidence that the presence of homing endonucleases appears to be common for *Felixounalikeviruses*.

Cluster 5 includes seven *Salmonella* phages, four phages sequenced in this study (i.e., FSL SP-031, FSL SP-038, FSL SP-049, and FSL SP-101, isolated from three different farms), two isolated in South Korea (i.e., SE2 and SS3e), and one isolated in the U.K. (i.e., SETP3) [43] (Figure 1A and Additional file 5)*.* Phages in this cluster have a genome size of approx. 43 kb (Table 1); 67 ORFs were predicted among these phage genomes, including 43 annotated as hypothetical proteins and 24 that were functionally annotated (Additional file 2). Cluster 5 represents two groups of phages (see Figure 1A); group 1 represents three of the phages sequenced here (FSL SP-031, FSL SP-038, and FSL SP-049); these three phages are highly conserved (78 to 100% aa identity among 64 shared orthologs). The other group (group 2) contains three previously sequenced phages and one of the phages sequenced here (SETP3, SS3e, SE2, and FSL SP-101) (Additional file 5). Group 1 phages FSL SP-031 and FSL SP-038 have 96% aa identity among 67 shared orthologs; the major differences between these two phages and FSL SP-049 are insertions and deletions of six hypothetical proteins and one homing endonuclease. This suggests that phages in this cluster are also hosts for homing endonucleases [40,42]. Each of the 7 phages in cluster 5 encodes a DNA/RNA helicase, which is a protein described to be involved in DNA replication and recombination [44] and could thus facilitate genomic rearrangements in phages in this cluster (Additional file 5). Overall, each of the four phage clusters discussed above contains not only phages from multiple farms, but also phages from at least two continents. In addition, two of these clusters contain the genomes of *Salmonella* phages that were isolated >50 years ago. Our findings here of closely related phages that are globally distributed are consistent with other genomic studies [22,37,45]; for instance, closely related *Pseudomonas* phages (up to 87% DNA identity) were isolated from different countries in Europe and the U.S. [46,47]. Similarly, closely related Mycobacteriophages were isolated from up to four different continents (i.e., North America, Europe, Asia and Africa) [48,49]. Interestingly, some of these phage groups contain closely related phages that appear to have adapted to different hosts, including hosts representing different bacterial genera. While a number of studies suggest rapid diversification of phages, e.g., through recombination [50,51], our data suggest that at least some phage groups are relatively stable at the genomic level and also suggest that phage dispersal could contribute to global spread of transmissible genomic elements, including those encoding resistance and putative virulence genes.

### Identification of phages carrying putative antimicrobial resistance and virulence genes

Virulence genes have been reported in a number of phages, including *E. coli* shiga toxigenic phages [52], and prophages, including the *Salmonella* prophages Gifsy-1 and Gifsy-2 [53-56]. In this study we describe the presence of putative antimicrobial resistance and virulence genes in genomes of virulent and temperate *Salmonella* phages. In the genome of FSL SP-016 we identified (i) one putative antimicrobial resistance gene, a homologue of *bcr*, which encodes a protein linked to bicyclomycin resistance. *bcr* has been reported in a number of different Gram-negative and positive bacteria. Bicyclomycin is an antimicrobial compound, which inhibits the transcription terminator factor Rho [57]. This antimicrobial is obtained from cultures of *Streptomyces* spp. and is effective against Gram-negative bacteria, including *Salmonella*[58]*.* Bicyclomycin is used as feed additive in livestock in some countries, but this use of bicyclomycin is not approved in the U.S. [58]. However, this antimicrobial could be synthesized naturally by *Streptomyces* spp., which are commonly found in soil [59]. As FSL SP-016 is a temperate phage, presence of phage-encoded bicyclomycin resistance could be a selective advantage for the phage as it could facilitate survival of the host; however, we have no evidence for horizontal gene transfer or lysogen expression that would lead to phenotypic bicyclomycin resistance; further experiments are necessary to determine if the presence of *bcr* can lead to phenotypic resistance.

In addition, each of the two phages in cluster 3 was found to carry (i) a putative virulence gene (a *yadA* homologue; 54% aa identity to *Enterobacter* sp. *yadA*) and (ii) a homologue of *terB* (67% aa identity with *E. coli terB*), which is part of a tellurite resistance operon (*ter*ZABCDEF) that has been found in the chromosome and plasmids of different bacteria [60,61] (Figure 2A & Table 2). Similarly, a TerZ homolog was reported in *Cronobacter* phage vB_CsaP_GAP52 (GenBank acc. No. NC_019402). *yadA* was first identified in enteropathogenic *Yersinia* species [62] where it was reported to encode an agglutinating adhesin (YadA, *Yersinia* adhesion), a virulence factor that has been reported to mediate *Yersinia* adherence to epithelial tissue [62]. In addition to the phages reported here, a *yadA* homologue has also been identified in Enterobacteria phage ΦEco32, *Synechococcus* phage metaG-MbCM1, *Prochlorococcus* phage P-HM2, and *Pectobacterium* phage My1; an alignment of ΦEco32 and the two phages of cluster 3 showed that they share no other regions of homology. Importantly, the genes encoding YadA homologs in FSL SP-058 and FSL SP-076 do not seem to locate close to genes encoding tail-morphogenesis proteins; rather, these genes are located next to a gene encoding a hypothetical protein and a gene encoding a deoxyuridine 5′-triphosphate nucleotide hydrolase. As both phages in cluster 3 did not contain a known lysogeny module, indicating that they are virulent phages, and did not show evidence for transduction (Table 3), it remains to be determined though if *yadA* can be transferred to *Salmonella* (or potentially other hosts) and if so if it would contribute to virulence in a recipient. As the function of *terB* remains unknown [60] and as only one gene from the *ter*ZABCDEF was present in the cluster 3 genomes, it is difficult to assess the functional importance of carrying this gene, even though tellurite is known to be toxic for bacteria because it generates reactive oxidative species (ROS), which can damage metabolic enzymes and cause lipid peroxidation [63]. Importantly, these two phages were isolated from two different farms, suggesting a certain selective pressure to maintain *terB*, and the agglutinating adhesin.

**Table 2:** Antimicrobial resistance, virulence and DNA metabolism genes identified in phages sequenced in this study

**Function**	**Gene**	**Phages harboring this gene**
Resistance	Tellurite resistance, TerB	Cluster 3
	Bicyclomycin resistance protein	FSL SP-016
Virulence	Agglutinating adhesin	Cluster 3
	Virulence protein MsgA/putative damage inducible protein DinI^1^	FSL SP-016
DNA metabolism	Thymidylate synthase	Clusters 2, 3, 4
	Ribonucleotide reductase of class III, large subunit	Clusters 2, 3, 4
	Ribonucleotide reductase of class III, activating subunit	Clusters 2, 3, 4
	Glutaredoxin	Clusters 2, 4
	Deoxyuridine 5′-triphosphate nucleotidohydrolase	Clusters 3, 6
	Dihydrofolate reductase	Cluster 4
	Ribose-phosphate pyrophosphokinase	Cluster 4
	Ribonucleotide reductase of class Ia, alpha subunit	Cluster 4
	Ribonucleotide reductase of class Ia, beta subunit	Cluster 4
	Exodeoxyribonuclease	Cluster 4
	Ribose phosphate pyrophosphokinase	Cluster 4
	Nicotinamide phosphoribosyltransferase	Cluster 4
	Deoxynucleotide monophosphate kinase	Cluster 4
	Ribonuclease HI	Cluster 2
	dCMP deaminase	Cluster 2
	Nucleoside 2-deoxyribosyltransferase	Cluster 1

^1^ This protein shows homology with both DinI ((pfam06183 [1.35e-08]) and with MsgA, (97% of aa identity with Gifsy-2 prophage MsgA). Experimental data will be needed to assign a functionally appropriate name to this gene.

**Table 3:** **Transduction frequency of chloramphenicol resistance for sequenced *Salmonella *phages that infected *S. *Typhimurium donor and recipient**

	**Transduction frequency**^**a**^**in Replicate 1**			**Transduction frequency**^**a**^**in Replicate 2**		
***Salmonella*Phage**	**MOI 0.1**	**MOI 1**	**MOI 10**	**MOI 0.1**	**MOI 1**	**MOI 10**
FSL SP-019	-	-	-	-	-	-
FSL SP-030	-	-	2.0 × 10^-6^	-	-	-
FSL SP-039	-	-	-	-	-	-
FSL SP-088	-	2.4 × 10^-5^	4.5 × 10^-6^	-	-	-
FSL SP-099	-	-	-	-	-	-
FSL SP-124	-	-	-	-	-	-
FSL SP-063	-	4.0 × 10^-6^	7.0 × 10^-7^	1.0 × 10^-5^	4.0 × 10^-6^	1.2 × 10^-6^
FSL SP-029	-	-	9.0 × 10^-7^	2.0 × 10^-5^	7.0 × 10^-6^	8.0 × 10^-7^
FSL SP-058	-	-	-	-	-	-
FSL SP-076	-	-	-	-	-	-
FSL SP-101	-	-	-	-	-	-

^a^ Transduction frequency was calculated as the number of transductants/PFU used for transduction; *MOI* multiplicity of infection.

In addition to carrying virulence and resistance genes, phages can also contribute to the dispersal of genes through lysogenic conversion and horizontal gene transfer [53]. We thus performed transduction assays, which found that 4/11 tested phages were able to transfer the chloramphenicol resistance gene from the *S.* Typhimurium donor strains to the recipient strain (see methods for details) (Table 3). The transduction frequency (ratio of transductants to PFU) [64] ranged from 9.0 × 10^-7^ to 2.4 × 10^-5^ (Table 3), which falls in the range previously reported for *Salmonella* phages. For example, *Salmonella* phage P22 had previously been shown to transduce at a frequency of 10^-7^ to 10^-4^[64,65]. Variations in transduction frequencies with different multiplicity of infections (MOI) and between replicates (Table 3) are consistent with previous reports [65,66]; for example, P22 has shown up to 1,000-fold differences in transduction frequency [65]. Interestingly, transducing phages belonged to two clusters, one identified in this study as representing temperate phages (cluster 1) and one previously identified as representing virulent phages (cluster 2). While transduction in temperate phages is easier to identify in laboratory conditions than in virulent phages (as virulent phages often lyse most of the transductants), a number of virulent phages have been identified as transducing phages [66]. This indicates that even if genome sequences indicate a virulent life cycle (e.g., absence of a known lysogeny module) and absence of phage-borne virulence and resistance genes, *in vitro* transduction tests are needed before a phage can be considered as safe to use as biocontrol agent.

### Identification of DNA metabolism genes on *Salmonella* phages with G+C contents different from *Salmonella*

In five phage clusters (cluster 1, 2, 3, 4 and 6) we identified a number of genes involved in DNA metabolism. All of these clusters represented phages with a G+C content different from the *Salmonella* genome (G+C content of approx. 50-52%); phages in clusters 1, 2, 3, 4, and 6 showed average G+C contents of 56%, 45%, 39%, 39%, and 42%, respectively (Table 1). However, in phages with a G+C content like *Salmonella* (cluster 5, cluster 8, and FSL SP-016, G+C content of 51%, 52%, 50%, respectively), no ORFs were annotated as DNA metabolism genes. The number of DNA metabolism genes ranged from 1 to 12 genes per phage. For example, all phages in cluster 4 (i.e., *Felixounalikeviruses*) carry 12 genes annotated as playing a role in DNA metabolism (Table 2). The same DNA metabolism genes had previously been identified in phages FelixO1 and ΦEa21-4 [40,41], suggesting that these genes are part of the genomic backbone of this phage genus. Phages in cluster 2 included six DNA metabolism genes (Table 2). These genes also appear to be part of the backbone of this phage cluster as supported by identification of these genes in closely related phages (e.g., Vi1, SFP10; see Figure 4A) that also grouped in this cluster [12,36,38,39]. DNA metabolism genes have previously been reported in a number of phages [12,21,36,38,40,67]; the best characterized phage carrying DNA metabolism genes is bacteriophage T4 [67,68]. In T4, proteins involved in DNA metabolism, replication, and repair function in a complex called T4 nucleotide precursor complex [67,68], which converts cellular nucleotide precursors into deoxynucleotide triphosphates with a different G+C ratio than the host [67]. As T4 has a G+C content of 34.5%, it uses the nucleotide precursor complex to adjust the host’s nucleotide ratios (approx. 50% G+C) to ratios needed for T4 replication [67]. Our data indicate that similar mechanisms may be at play to facilitate replication of *Salmonella* phages whose G+C content differ from their host. Expression of DNA metabolism genes may thus be a common mechanism used to debottleneck DNA replication in some phages with a G+C content different from their hosts.

### Evidence of *Salmonella* phage tailspike and fiber diversity due to both single nucleotide polymorphisms and major re-arrangements

Tail fibers and tailspikes are appendages in the phage tail that facilitate the initial binding of the phage to the bacterial host; they hence represent primary receptor-binding proteins. Phages can have one or more tailspikes or fibers, or combinations of tailspikes and fibers [69]. These virion structures target proteins or polysaccharides in the host surface, and therefore, have a role in host specificity [69,70]; nucleotide polymorphisms, duplications and rearrangements in the tail fiber and tailspike encoding genes appear to be associated with changes in the host range [30]. As our initial analysis (see above) found evidence for (i) major re-arrangements in genes encoding tailspikes and fibers in some orthoclusters (clusters 2, 5, and 3) and (ii) small number of SNPs in the tail fibers of the three other orthoclusters (clusters 1, 4, and 6), we performed more detailed analyses to characterize the genetic diversity in genes encoding tailspikes and fibers.

In the six phages in orthocluster 2, the region that contains the four genes encoding tailspikes is by far the most variable region of the genome (Figure 5A & Additional file 6); however, the tail fiber gene is conserved among all six phages (pairwise aa identities ranged from 66 to 97%; Additional file 6). In addition, for two phages in this cluster (FSL SP-029 and FSL SP-063, isolated from different farms), overall aa identities in all tailspikes and the tail fiber were high (97-100%; Figure 5A). Highly variable tailspikes typically presented a fairly conserved N-terminal, with low aa identities in the C terminal (10 to 37%, except for phages FSL SP-029 and FSL SP-063) (Figure 5A). These findings are consistent with Hooton et al. [12], who previously analyzed the tailspikes in three phages of the *Viunalikevirus* genus (ΦSH19, Vi1, ΦSbom-AG3); conservation of the N-terminal residues is also consistent with the fact that this region attaches to the baseplate, while the remainder of the protein projects from the surface and appears to be involved in initial binding to the host [12,69]. Although future experimental work is needed, a role of the C-terminal variable regions in phage host specificity is supported by the observation that one of the tailspikes of the *Salmonella* Typhi specific phage Vi1 has a conserved acetyl esterase domain that recognizes the Typhi capsule as receptor [38]. Phage SFP10, which was reported to infect both *Salmonella* and *E. coli* O157:H7, also carries tailspikes that presented similarity to tailspikes found in both *E. coli* and *Salmonella* phages [39], suggesting a possible role of these regions in host specificity.

Phages in orthocluster 3 (FSL SP-058 and FSL SP-076) encoded two tailspikes, these proteins also presented a conserved N-terminal and a highly divergent C-terminal (7 and 33% aa identity) (Figure 5B); these two phages show considerable differences in host range (2 and 3 *Salmonella* host strains are only lysed by FSL SP-058 and FSL SP-076, respectively; see Additional file 7). While the two tail fibers in the orthocluster 5 phages typically show a higher level of conservation and a longer conserved N-terminal region, the C-terminal region of one tail fiber (tail fiber 1) still shows considerable variability in four of these phages (Figure 5C & Additional file 6). The second tail fiber gene in cluster 5 presented two distinct alleles; phages FSL SP-031, FSL SP-038 and FSL SP-049 share one conserved tail fiber (99 to 100% aa identity) as do phages Se2, SEPT3, SS3e, and FSL SP-101 (94 to 95% aa identity), with only 27% average aa identity between these two tail fibers (see Additional file 6).

Phages in orthoclusters 1, 4, and 6, on the other hand, showed low divergence in their tail fibers. For example, among the four phages in orthocluster 4, tail fiber 1 was highly conserved (96 to 100% aa identity, Figure 5E), while tail fiber 2 showed 81 to 93% aa identity among these phages (Figure 5E). Interestingly, these phages differ considerably in their host range; FelixO1 infected all 23 *Salmonella* strains tested (in a previous report [71] FelixO1 lysed 98.2% of *Salmonella* isolates tested), while FSL SP-010, FSL SP-012, and FSL SP-107 present a narrower host range infecting 4, 7 and 3 strains, respectively (see Additional file 7). Further experimental and comparative genomics studies of these phages may thus provide an opportunity to understand why some phages such as FelixO1 have an extremely wide host range. The two phage variants in cluster 6 showed 100% aa identity across the proteins they encode except in the tail fiber, which presented three amino acids changes (Figure 3B); two substitutions of glutamic acid to glycine and one of phenylalanine to isoleucine (Figure 3B). While these two phage variants were isolated from the same farm, FSL SP-062 infects *S.* Newport and *S.* Kentucky, but FSL SP-069 only infects *S.* Newport (Additional file 7). Experimental studies are necessary to validate the role of these mutations in the increase of FSL SP-062 host range. Further studies on the contributions of different diversification patterns on phage host specificity, facilitated by the comparative genomics studies reported here, will provide a possibility to advance towards a more rational approach to constructing both narrow and wide host range *Salmonella* phages, which may open new opportunities in phage based detection and biocontrol.

## Conclusions

This study used a genomic approach to investigate the diversity of 22 newly sequenced *Salmonella* phages. A number of novel *Salmonella* phages were identified, representing phages different from currently known phage genera infecting *Enterobacteriaceae*. Comparative genomics analyses of these phages have provided a number of new insights in the evolution and diversity of *Salmonella* phages, such as identification of a possible wide-spread role of DNA metabolism genes in facilitating replication of phages with GC contents distinct from the GC content of *Salmonella*. Our data also elude to the possibility of distinct mechanisms, among different phage families, of tail fiber and tailspike diversification, which may be linked to evolution of host specificity. Clearly, our analyses reported here only provide a first glimpse into the diversity of *Salmonella* phages that is likely to be discovered when phages from different environments are characterized by full genome sequencing and metagenomic approaches.

## Methods

### Phage isolates

A total of 22 phages were selected for whole genome sequencing (Table 1). These phages were previously isolated and characterized, including for host range and genome size, by pulsed field gel electrophoresis [7]. According to these previously characterized features, these phages were selected to represent diversity within our collection, including phages with narrow and wide host range, phages that infect different *Salmonella* serovars, and phages isolated from different farms in New York State. Phage lysate preparation and DNA extraction was conducted as previously described [7,72]. Briefly, DNA extraction was performed with phenol/chloroform, followed by ethanol precipitation. DNA was dissolved in 50-100 μl of TE buffer (10 mM Tris, 1 mM EDTA; pH 8.0) and quantified using OD_260_ values measured with a Nanodrop Spectrophotometer (NanoDrop products, Wilmington, DE).

### Sequencing and annotation workflow

Phage genomes were sequenced with the Illumina Genome Analyzer IIx (Illumina Inc. San Diego, CA) at the Cornell University Life Sciences Core Laboratories Center. Fifty-base pair reads were assembled *de novo* using the Velvet algorithm [73]. For 11 phages, the genome was assembled into one single contig; in phages with multiple contigs a pseudogenome was prepared for comparison purposes. Briefly, contigs were ordered according to their alignment to a reference phage (phage that presented homology among the phages sequenced in this study or previously sequenced phages). Then, contigs were merged with a pseudomarker (nnn), which was added to identify the different contigs. Contigs were annotated using a combination of automatic annotations by RAST [74], and the NCBI Prokaryotic Genomes Automatic Annotation Pipeline (PGAAP) [75], followed by manual curation using RAST. Sequences are available in Additional file 8, and pseudogenomes are available at the Cornell Food Safety Laboratory Microbial Genome Data Wiki page (https://confluence.cornell.edu/display/FOODSAFETY/Cornell+Food+Safety+Laboratory+Microbial+Genome+Data) [76]. A summary of annotations for the phages sequenced here is available in Additional file 2.

### Clustering and comparative analysis

To classify phages into clusters, an orthologous gene presence/absence matrix was created using OrthoMCL v.1.4 [77] with the default settings. This matrix with the presence and absence of predicted proteins was used to prepare a neighbor-joining tree using Splits Tree4 [78]. This cluster analysis included genomes of (i) 20 non-redundant phages that represent the type species of all known phage genera infecting the *Enterobacteriaceae*[25], (ii) 23 previously reported *Salmonella* phages (representing all *Salmonella* phages available in http://www.ebi.ac.uk/genomes/phage.html as of August, 2012), and (iii) 22 phages reported and sequenced here (Additional file 1). Genome-wide nucleotide comparisons were performed using Gepard 1.3 [79] and the concatenated genome sequences of the 22 phages sequenced here. To facilitate the display of the nucleotide similarities as a dot plot, phage genomes were ordered according to their placement in the previously identified orthoclusters.

Whole genome alignments were conducted with Mauve [80], and the guide tree was used to visualize overall phages similarity. Comparisons were conducted within clusters with Mauve and RAST. Nucleotide and amino acid sequence alignments, and pairwise comparisons were conducted in MegAlign (DNASTAR Inc, Madison, WI), with the ClustalW algorithm. Linear representations of the whole genome BLAST comparisons were produced with Easyfig [81].

For each phage, all predicted ORFs were searched against GenBank using the BLASTP algorithm [82]. Hits in this search were used to identify similar phage genomes already in GenBank; similarity with previous phages was also used to aid with classification of newly sequenced phages into putative phage families. Phages in which a known lysogeny module was annotated were classified as putative temperate phages, phages without a known lysogeny module were classified as putative virulent phages [38,39].

### Bacteriophage mediated-transduction

Transduction assays were conducted as described for *Salmonella* phage P22 [65]. These assays were conducted only on phages that infected *S.* Typhimurium donor and recipient strains (Table 3). For donor strain we used *S.* Typhimurium FSL R8-3980, which has a chromosomally inserted *cat* gene (chloramphenicol resistance); as recipient we used the wild type strain FSL R8-3981. A phage lysate was prepared using the donor strain; for this, 200 μl of an overnight culture of the donor, a phage concentration of 5 × 10^6^ PFU/ml, and 1 ml of LB broth (Bacto, Franklin Lakes, NJ) were mixed and incubated for 12–16 h at 37°C. Phage lysate was recovered by adding 4–5 drops of chloroform, followed by centrifugation for 10 min at 10,000 rpm; the lysate was then titered. For transduction, this lysate was used to infect the recipient. Briefly, an overnight culture of the recipient was diluted 50-fold into LB broth, and incubated at 37°C to log phase (1.5-3 h), phage was then added in three different multiplicity of infections (i.e., 0.1, 1, and 10), and lysate was incubated at 37°C for 30 min. To avoid secondary infections 0.5 ml of 20 mM EGTA (Fisher scientific, Pittsburgh, PA) was added, and lysate was incubated for 1 h at 37°C with shaking. Samples were centrifuged, and pellet was suspended in 100 μl of LB broth. All 100 μl were then spread on plates prepared with 20 μg of chloramphenicol (Sigma-Aldrich, St. Louis, MO) and 10 mM EGTA, followed by an overnight incubation at 37°C. Positive control was phage P22, and negative control was sterile water; both controls were used in all the steps. Transduction frequencies were calculated as the ratio of the number of transductants/PFU added for transduction [65,83]. Experiments were conducted in two independent replicates; if transduction was observed in at least one of the replicates, this phage was classified as a transducing phage.

### Availability of supporting data

The sequence data supporting the results of this article are available on GenBank under the following accession numbers [GenBank: KC139511 to GenBank: KC139680].

## Competing interests

The authors declare that they have no competing interests.

## Authors’ contributions

AIMS, CA, MW: designed experiments; AIMS: conducted experiments; HB: participated in the sequences assembly; AIMS, RHO, HB, KV, MW: participated in genome analysis, and data interpretation; AIMS, MW: wrote the manuscript. All authors read and approved the final manuscript.

## Supplementary Material

Additional file 1 **List of previously sequenced *Salmonella * phages used for comparative analysis.** PDF file containing the list of previously sequenced phages used for comparative analysis. Click here for file

Additional file 2 **Summary of annotations in the different phages.** PDF file containing a summary of functionally annotated ORFs in the phages sequenced here. Click here for file

Additional file 3 **Comparison, using the BLAST algorithm, of novel phages in cluster 1.** PDF file containing the comparison of phages in cluster 1. Phages and farm origins are in the right side of the figure. Orange arrows indicate open reading frames (ORFs); grey shaded regions indicate regions with homology. Click here for file

Additional file 4 **Comparison, using the BLAST algorithm, of FSL SP-088 and *Enterobacter *phage Enc34.** PDF file containing the comparison using the BLAST algorithm of FSL SP-088 and *Enterobacter* phage Enc34. Orange arrows indicate ORFs, and regions of homology are shaded in grey. Click here for file

Additional file 5 **Comparison, using the BLAST algorithm, of phages in cluster 5.** PDF file containing the comparison of phages in cluster 5, representing four phages sequenced in this study (FSL SP-031, FSL SP-038, FSL SP-049 and FSL SP-101) and three phages previously sequenced in South Korea and U.K. Phages and country of origin are indicated on the right, purple arrows indicate ORFs, and grey shaded regions are regions of homology. On the left side is the tree generated with the Mauve algorithm indicating the overall similarity of the phages, this tree identified two branches named as 1 and 2. Click here for file

Additional file 6 **Pairwise comparison of the amino acid identities on tailspikes and fibers.** PDF file containing the percentage of amino acid identity of tailspikes and fibers in phage clusters. Click here for file

Additional file 7 **Host range of sequenced phage clusters.** PDF file containing the host range of sequenced phages in clusters. Click here for file

Additional file 8 **Sequence data.** Text file containing gbk files of all 22 sequenced phage genomes. Click here for file
