# Supplementary material for: Genomic characterization provides new insight into Salmonella phage diversity
Source: BMC Genomics. 2013 Jul 17;14:481. doi: 10.1186/1471-2164-14-481 (PMC3728262; doi:10.1186/1471-2164-14-481)
Supplement: Additional file 1 — List of previously sequenced Salmonella phages used for comparative analysis. PDF file containing the list of previously sequenced phages used for comparative analysis. [file 1471-2164-14-481-S1.pdf]

Additional file 1. List of previously sequenced phages for clustering and comparative analysis

Salmonella Phage	host	Isolation origin	Size (bp)	Accession No.	Publication
**Previously sequenced *Salmonella* phages used for clustering and comparative analysis[1]**					
phage 7-11	*S.* Newport	Canada	89,916	NC_015938	21107618
phage ε34	*S.* Anatum	U.S.	43,016	NC_011976	17113244
phage g341c	*Salmonella*	U.S.	40,975	FJ000341	Unpublished
phage HK620	*Salmonella*	U.S.	38,297	AF335538	11518522
phage ΦSG-JL2	*S.* Gallinarum	South Korea	38,815	NC_010807	18820072
phage ΦSH19	*S.* Typhimurium	UK	157,785	JN126049	22047448
phage RE-2010	*Salmonella*	U.S.	34,117	HM770079	Unpublished
phage SE2	*Salmonella*	South Korea	43,221	NC_016763	2273387
phage SFP10	*S.* Typhimurium	South Korea	157,950	NC_016073	22020516
phage SPN1S	*S.* Typhimurium	Spain	38,684	NC_016761	22205721
phage SPN3US	*S.* Typhimurium	South Korea	240,413	JN641803	22106383
phage SS3e (KS5)	*S.* Typhimurium	South Korea	40,793	NC_006940	22923809
phage ST64B	*S.* Typhimurium	Australia	40,149	AY055382	14563886
phage ST160	*S.* Typhimurium	New Zealand	40,986	NC_014900	20950514
phage E1	*S.* Typhi	UK	45,051	AM491472	18192390
phage Vi06	*S.* Typhi	UK	38,368	NC_015271	20817773
phage Fels-2	*S.* Typhimurium	U.S.	33,693	NC_010463	11677609
phage ST104	*S.* Typhimurium	Japan	41,391	NC_005841	15071057
phage Fels-1	*S.* Typhimurium	U.S.	42723	NC_010391	11677609
Phage Gifsy-2	*S.* Typhimurium	U.S.	45,840	NC_010393	11677609
Phage FO1a	*Salmonella*	Switzerland	83,331	JF461087	Unpublished
Phage SPN19	*Salmonella*	South Korea	59,203	JN871591	22927964
Phage PVP-SE1	*Salmonella*	U.S.	145,964	NC_016071	21865376
**Other phages used for comparative analysis**					
phage EC6	*E. coli*	South Korea	86,231	JX560968	23405293
phage SPT-1	*Salmonella*	U.S.	86,626	JX181822	15982775
Phage wV8	*E. coli*	Canada	88,487	NC_012749	19379502
Phage ΦSboM-AG3	*Shigella boydii*	Canada	158,006	NC_013693	21595934
Phage ΦEa21-4	*Erwinia*	U.S.	84,576	NC_011811	19181832
Phage Enc34	*Enterobacter*	Latvia	60,364	JQ340774	22997422
**Type species of phage genera infecting *Enterobacteriaceae* used for clustering and comparative analysis[2]**					
**Phage**	**Family**	**genus**	**size**	**GenBank acc. No.**	**publication**
Phage phiO18P	*Myoviridae*	*Hpunalikevirus*	33,985	NC_009542	18096197
Phage P2	*Myoviridae*	*P2likevirus*	33,593	NC_001895	12426340
Phage T4	*Myoviridae*	*T4likevirus*	168,903	NC_000866	12626685
Phage FelixO1	*Myoviridae*	*Felixounalikevirus*	86,155	AF320576	21994654
Phage Mu	*Myoviridae*	*Mulikevirus*	36,717	NC_000929	11922669
Phage P1	*Myoviridae*	*Punalikevirus*	94,800	NC_005856	15489417
Phage ViI	*Myoviridae*	*Viunalikevirus*	157,061	NC_015296	20817773
Phage SP6	*Podoviridae*	*Sp6likevirus*	43,769	NC_004831	15028677
Phage T7	*Podoviridae*	*T7likevirus*	39,937	NC_001604	8171031
Phage epsilon15	*Podoviridae*	*Epsilon15likevirus*	39,671	NC_004775	17825342
Phage N4	*Podoviridae*	*N4likevirus*	70,153	NC_008720	12193610
Phage P22	*Podoviridae*	*P22likevirus*	41,724	NC_002371	12562822
Phage Phieco32	*Podoviridae*	*Phieco32likevirus*	77,554	NC_010324	18294652

Phage KP34	*Podoviridae*	Phikmvlikevirus	43,809	NC_013649	21327407
phage lambda	*Siphoviridae*	*Lambdalikevirus*	48,502	NC_001416	3038914
phage N15	*Siphoviridae*	*N15likevirus*	46,375	NC_001901	8631680
phage T5	*Siphoviridae*	*T5likevirus*	121,750	NC_005859	9929878
phage T1	*Siphoviridae*	*Tunalikevirus*	48,836	NC_005833	14972552
phage HK578	*Siphoviridae*	*Hk578likevirus*	43,741	NC_019724	Unpublished
phage SETP3	*Siphoviridae*	*To be assigned*[3]	42,572	NC_009232	19074657

[1] Selected *Salmonella* phages available in http://www.ebi.ac.uk/genomes/phage.html as of August, 2012
[2] Non-redundant phages that represent the type species of all known phage genera infecting the *Enterobacteriacea* according to http://ictvonline.org/virusTaxonomy.asp?version=2012.
[3] Personal communications with Dr. Andrew Kropinski
