# Supplementary material for: Genomic characterization provides new insight into Salmonella phage diversity
Source: BMC Genomics. 2013 Jul 17;14:481. doi: 10.1186/1471-2164-14-481 (PMC3728262; doi:10.1186/1471-2164-14-481)
Supplement: Additional file 2 — Summary of annotations in the different phages. PDF file containing a summary of functionally annotated ORFs in the phages sequenced here. [file 1471-2164-14-481-S2.pdf]

Additional file 2. Summary of annotations in the different clusters

Cluster	Phages FSL	Structural proteins	Lysogeny module	Lysis module	Replication & recombination	DNA metabolism	Not related with phage life cycle
1	SP-019<br>SP-030<br>SP-039<br>SP088<br>SP-099<br>SP-124	Terminase small & large subunits, head-tail joining, portal protein, prohead protease ClpP, decorator protein D, capsid protein E, pre-tape measure, tape measure protein, tail assembly protein, tail fiber protein, virion protein	Cro/C1 protein, XRE-family of transcriptional regulators	Holin, endolysin	Primase, helicase, DNA polymerase, NinC, DNA adenine methylase,	Nucleoside 2-deoxyribosyl transferase,	-
2	SP-029<br>SP-063	baseplate wedge subunit, tail fibre, head completion protein, baseplate tail tube, base plate hub subunit, tail completion, major capsid protein, prohead core protein, prohead core scaffolding protein, portal vertex protein, tail tube protein, tail sheath protein, large terminase protein, tail sheath stabilizer and completion protein, neck protein, tail spike protein	-	lysozyme	DNA polymerase, RIIA, RIIB, DNA topoisomerase II, DexA exonuclease A, helicase, DNA ligase, DNA end protector protein, single-stranded DNA binding protein, DNA primase, recombination endonuclease, RNA polymerase sigma factor, ribonuclease H, DNA-binding HU protein, clamp loader subunit, sliding clamp UvsW helicase, GIY-YIG endonuclease exonuclease	dUTP diphosphatase, thymidylate synthase, ribonucleotide reductase of class III large subunit, ribonucleotide reductase of class III activating subunit glutaredoxin, dCMP deaminase, ribonuclease HI	-
3	SP-058<br>SP-076	major coat protein, tail spike protein, phage-related terminase	-	Holin, phage lysis protein	RNA polymerase, NinI protein, DNA helicase, DNA polymerase, single-strand DNA	dUTPase, thymidylate synthetase, ribonucleotide reductase of class III large subunit,	agglutinating adhesin, Tellurite resistance

					binding protein	ribonucleotide reductase of class III activating subunit, deoxyuridine 5'-triphosphate nucleotidohydrolase	protein
8	SP-004	antitermination protein Q, portal vertex protein, terminase ATPase subunit, capsid-scaffolding protein, capsid protein, terminase endonuclease subunit, tail protein, baseplate assembly protein, baseplate wedge subunit, baseplate assembly protein, tail fibre protein, major tail sheath protein, major tail tube protein, Phage protein U, Phage protein D	Integrase, transcriptional regulator, Cox protein	Lysin, LysB, LysC	replication protein, DksA-like zinc finger domain containing protein	-	-
-	SP-016	tail fiber, tail tape measure protein, major tail subunit, head-tail adaptor, portal protein, head maturation protease, phage terminase-like protein, phage terminase, small subunit, antitermination protein Q	regulatory protein CII, regulatory protein CI, regulatory protein Cro, integrase, excisionase	endolysin	exodeoxyribonuclease VIII, RecT, site-specific recombinases, restriction endonuclease, NinG, DNA replication protein	-	Bicyclomycin efflux system
9	SP-126	tail fiber protein, tail component, minor tail protein, major tail protein, major capsid protein, prohead protease, phage head morphogenesis, portal protein, terminase large subunit, terminase	-	LysN, holin	Single strand binding protein, recombination protein, exodeoxyribonuclease, polynucleotide kinase/phosphatase, helicase, DNA primase, Dam methylase	-	-

		small subunit					
4	SP-010<br>SP-012<br>SP-107	tail fiber protein, baseplate component, baseplate assembly protein, major capsid protein, prohead protease, terminase large subunit, tail protein		Lysin	DNA ligase, DNA polymerase, endonuclease, DNA primase/helicase, exodeoxyribonuclease, rIIA protein, rIIB protein	Thymidylate synthase, dihydrofolate reductase, deoxynucleotide monophosphate kinase, ribonucleotide reductase of class III large subunit, ribonucleotide reductase of class III activating subunit, glutaredoxin, deoxyuridine 5'-triphosphate nucleotidohydrolase, ribose-phosphate pyrophosphokinase, ribonucleotide reductase of class Ia alpha, ribonucleotide reductase of class Ia beta, exodeoxyribonuclease, ribose phosphate pyrophosphokinase, nicotinamide phosphoribosyl transferase	HNH endonuclease
5	SP-031<br>SP-038<br>SP-049<br>SP-101	head morphogenesis protein, coat protein, tail length tape measure protein, tailspike protein	-	lysozyme	Helicase, DNA polymerase I, DNA modification protein, helicase/primase	-	HNH endonuclease
6	SP-062<br>SP-069	Terminase TerL, head morphogenesis protein, capsid related protein	-	endolysin	RdgC exonuclease, endodeoxyribonuclease, primase, ParB-like nuclease domain	deoxyuridine 5'-triphosphate nucleotidohydrolase	-
