# Supplementary figures and images for: Genomic characterization provides new insight into Salmonella phage diversity
Source: BMC Genomics. 2013 Jul 17;14:481. doi: 10.1186/1471-2164-14-481 (PMC3728262; doi:10.1186/1471-2164-14-481)

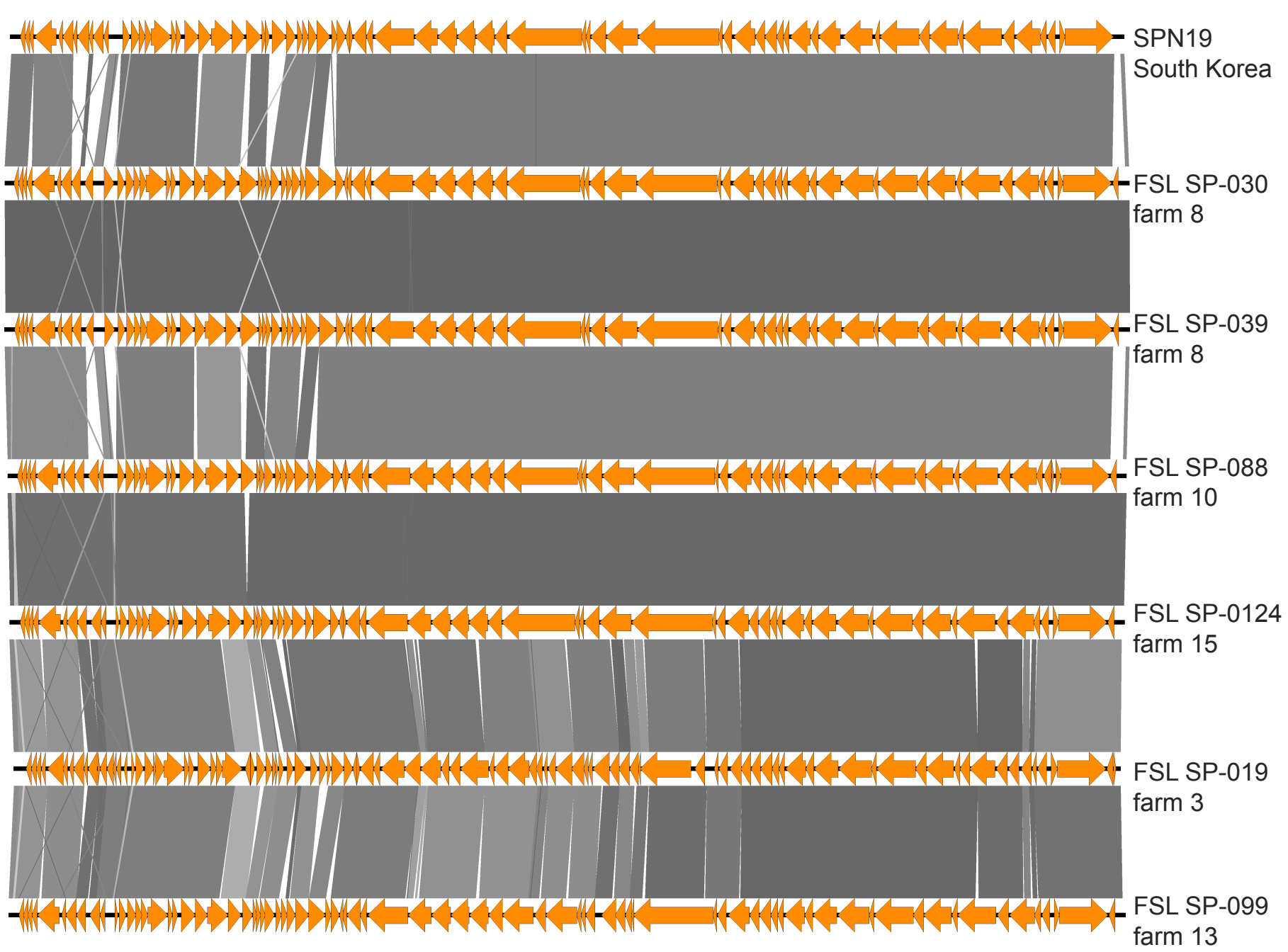

Supplement: Additional file 3 — Comparison, using the BLAST algorithm, of novel phages in cluster 1. PDF file containing the comparison of phages in cluster 1. Phages and farm origins are in the right side of the figure. Orange arrows indicate open reading frames (ORFs); grey shaded regions indicate regions with homology. [file 1471-2164-14-481-S3.pdf]

FSL SP-088

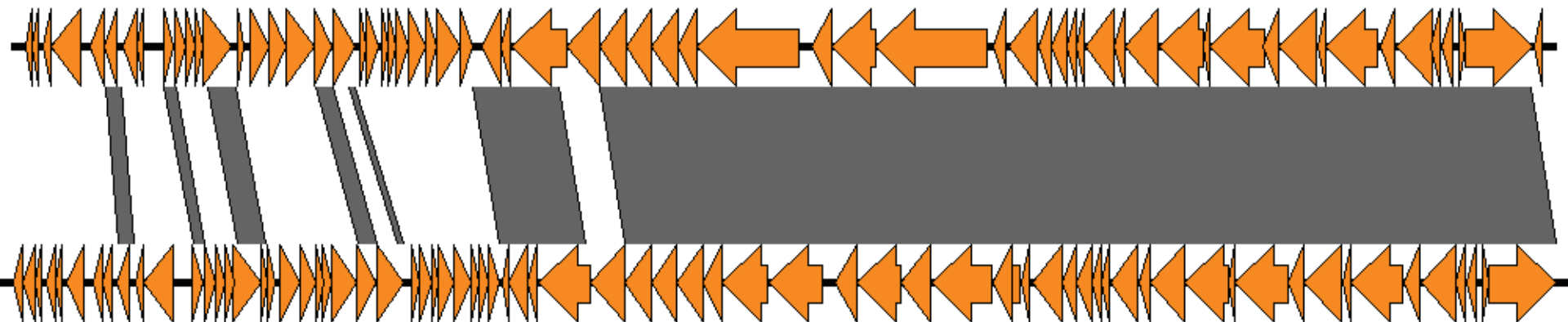

Enterobacter phage Enc34

Supplement: Additional file 4 — Comparison, using the BLAST algorithm, of FSL SP-088 and Enterobacter phage Enc34. PDF file containing the comparison using the BLAST algorithm of FSL SP-088 and Enterobacter phage Enc34. Orange arrows indicate ORFs, and regions of homology are shaded in grey. [file 1471-2164-14-481-S4.pdf]

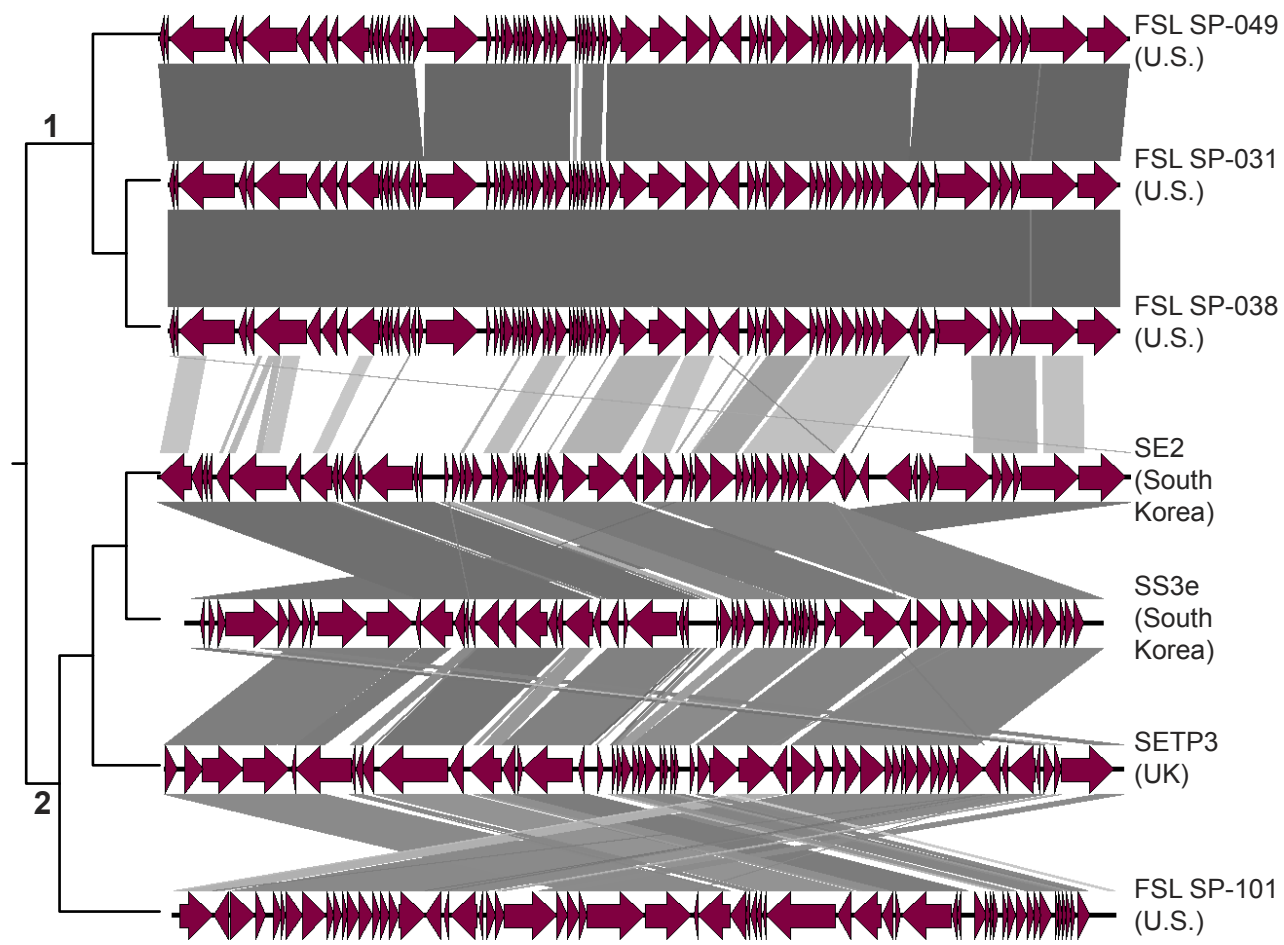

Supplement: Additional file 5 — Comparison, using the BLAST algorithm, of phages in cluster 5. PDF file containing the comparison of phages in cluster 5, representing four phages sequenced in this study (FSL SP-031, FSL SP-038, FSL SP-049 and FSL SP-101) and three phages previously sequenced in South Korea and U.K. Phages and country of origin are indicated on the right, purple arrows indicate ORFs, and grey shaded regions are regions of homology. On the left side is the tree generated with the Mauve algorithm indicating the overall similarity of the phages, this tree identified two branches named as 1 and 2. [file 1471-2164-14-481-S5.pdf]
