# Supplementary material for: Genomic characterization provides new insight into Salmonella phage diversity
Source: BMC Genomics. 2013 Jul 17;14:481. doi: 10.1186/1471-2164-14-481 (PMC3728262; doi:10.1186/1471-2164-14-481)
Supplement: Additional file 6 — Pairwise comparison of the amino acid identities on tailspikes and fibers. PDF file containing the percentage of amino acid identity of tailspikes and fibers in phage clusters. [file 1471-2164-14-481-S6.pdf]

Additional file 6. Pairwise comparison of the amino acid identities on tailspikes and fibers

High divergence

Cluster 2: Tail fiber 1

	PhiSbom			PhiSH19			SFP10			SP-029			SP-063			Vi01		
	total	con	unc	total	con	unc	total	con	unc	total	con	unc	total	con	unc	total	con	unc
PhiSbom	100	100	100	66.2	88.2	58.2	66.4	89.6	58.2	66.4	89.6	58.2	65.9	88.3	57.8	66.4	88.3	58.5
PhiSH19				100	100	100	99.2	97.4	99.7	97.5	97.4	97.5	99.2	98.7	99.4	93.2	98.7	92.0
SFP10							100	100	100	98.2	100	97.8	99.0	96.1	99.7	92.7	96.1	92.0
SP-029										100	100	100	97.7	96.1	98.2	90.9	96.1	89.8
SP-063													100	100	100	93.2	100	91.7
Vi01																100	100	100

Cluster 2: Tailspike 1

	PhiSH19			SFP10			SP-029			SP-063			Vi01		
	total	con	unc	total	con	unc	total	con	unc	total	con	unc	total	con	unc
PhiSH19	100	100	100	23.1	58.3	22.4	24.2	93.3	18.2	24.2	93.3	18.2	30.1	95.0	35.1
SFP10				100	100	100	21.9	63.3	15.5	21.9	63.3	15.5	22.5	61.7	20.4
SP-029							100	100	100	100	100	100	29.4	93.3	23.2
SP-063										100	100	100	29.4	93.3	23.2
Vi01													100	100	100

Cluster 2: Tailspike 2

	PhiSbom			PhiSH19			SFP10			SP-029			SP-063			Vi01		
	total	con	unc	total	con	unc	total	con	unc	total	con	unc	total	con	unc	total	con	unc
PhiSbom	100	100	100	26.4	73.8	26.2	24.2	73.0	10.5	24.5	74.5	10.5	24.5	74.5	10.5	25.4	76.6	26.0
PhiSH19				100	100	100	47.7	97.9	28.0	47.6	98.6	27.8	47.6	98.6	27.8	41.9	95.0	37.8
SFP10							100	100	100	98.3	96.5	98.6	98.3	96.5	98.6	39.3	92.9	20.9
SP-029										100	100	100	100	100	100	39.7	96.5	20.7
SP-063													100	100	100	39.7	96.5	20.7
Vi01																100	100	100

Cluster 2: Tailspike 3 (conserved in the all length)

	PhiSH19			SFP10			SP-029			SP-063		
	total	con	unc	total	con	unc	total	con	unc	total	con	unc
PhiSH19	100	100	100	97.7	-	-	98.1	-	-	98.1	-	-
SFP10				100	100	100	97.7	-	-	97.7	-	-
SP-029							100	100	100	100	-	-
SP-063										100	100	100

Cluster 2: Tailspike 4

	PhiSbom			PhiSH19			SFP10			SP-029			SP-063			Vi01		
	total	con	unc	total	con	unc	total	con	unc	total	con	unc	total	con	unc	total	con	unc
PhiSbom	100	100	100	32.0	67.5	24.5	31.6	66.9	15.3	29.8	67.9	11.8	31.2	67.9	11.9	34.5	69.5	27.2
PhiSH19				100	100	100	55.2	98.4	31.3	51.1	96.4	26.7	52.9	96.4	26.7	41.9	90.8	22.6
SFP10							100	100	100	45.0	97.4	23.7	46.8	97.4	23.7	39.8	89.2	17.1
SP-029										100	100	100	99.5	100	99.3	39.1	91.8	15.2
SP-063													100	100	100	41.2	91.8	15.2
Vi01																100	100	100

Cluster 3: Tailspike 1

	SP-058			SP-076		
	total	con	unc	total	con	unc
SP-058	100	100	100	29.5	60.1	6.9
SP-076				100	100	100

Cluster 3: Tailspike 2

	SP-058			SP-076		
	total	con	unc	total	con	unc
SP-058	100	100	100	37.9	72.8	13.1
SP-076				100	100	100

Cluster 5: tail fiber 1 (C-terminal conserved)

	Se2			SEPT3			SP-031			SP-038			SP-049			SP-101			SS3e		
	total	con	unc	total	con	unc	total	con	unc	total	con	unc	total	con	unc	total	con	unc	total	con	unc
Se2	100	100	100	98.0	99.0	28.6	72.3	70.7	74.8	72.3	70.7	74.8	72.3	70.7	74.8	98.5	99.2	96.9	99.0	99.3	6.8
SEPT3				100	100	100	70.9	71.2	28.6	70.9	71.2	28.6	70.9	71.2	28.6	98.2	99.2	28.6	97.7	98.6	28.6
SP-31							100	100	100	100	100	100	100	100	100	72.3	71.0	74.0	69.4	70.7	5.9
SP-38										100	100	100	100	100	100	72.3	71.0	74.0	69.4	70.7	5.9
SP-49													100	100	100	72.3	71.0	74.0	69.4	70.7	5.9
SP101																100	100	100	98.4	98.8	6.8
SS3e																			100	100	100

Cluster 5: tail fiber 2

	Se2			SEPT3			SP-031			SP-038			SP-049			SP-101			SS3e		
	total	con	unc	total	con	unc	total	con	unc	total	con	unc	total	con	unc	total	con	unc	total	con	unc
Se2	100	100	100	95.2	96.6	95.0	27.2	82.8	15.5	27.2	82.8	15.5	27.4	82.8	15.7	94.3	100	93.5	98.2	100	98.0
SEPT3				100	100	100	26.7	82.8	15.0	26.7	82.8	15.0	26.9	82.8	15.2	93.7	96.6	93.4	95.5	96.6	95.4
SP-31							100	100	100	100	100	100	99.7	100	99.7	27.4	82.8	15.7	27.4	82.8	15.7
SP-38										100	100	100	99.7	100	99.7	27.4	82.8	15.7	27.4	82.8	15.7
SP-49													100	100	100	27.6	82.8	15.9	27.6	82.8	15.9
SP101																100	100	100	94.4	82.8	93.7
SS3e																			100	100	100

Low divergence

Cluster 1: tail fiber

	SP-019			SP-030			SP-039			SP-088			SP-099			SP-124		
	total	con	unc	total	con	unc	total	con	unc	total	con	unc	total	con	unc	total	con	unc
SP-019	100	100	100	96.3	-	-	96.3	-	-	96.6	-	-	97.2	-	-	99.6	-	-
SP-030				100	100	100	100	-	-	95.9	-	-	97.6	-	-	95.9	-	-
SP-039							100	100	100	95.9	-	-	97.6	-	-	95.9	-	-
SP-088										100	100	100	96.7	-	-	99.2	-	-
SP-099													100	100	100	97.6	-	-
SP-124																100	100	100

Cluster 4: tail fiber 1

	Felix O1			SP-10			SP-012			SP-107		
	total	con	unc	total	con	unc	total	con	unc	total	con	unc
Felix O1	100	100	100	97.7	-	-	97.7	-	-	96.6	-	-
SP-010				100	100	100	100	-	-	96.2	-	-
SP-012							100	100	100	96.2	-	-
SP-107										100	100	100

Cluster 4: tail fiber 2

	Felix O1			SP-10			SP-012			SP-107		
	total	con	unc	total	con	unc	total	con	unc	total	con	unc
Felix O1	100	100	100	81.0	-	-	74.9	-	-	81.4	-	-
SP-010				100	100	100	92.9	-	-	90.7	-	-
SP-012							100	100	100	87.7	-	-
SP-107										100	100	100

Cluster 6: tail fiber

	SP-062			SP-069		
	total	con	unc	total	con	unc
SP-062	100	100	100	99.5	-	-
SP-069				100	100	100
