# Supplementary material for: Genomic characterization provides new insight into Salmonella phage diversity
Source: BMC Genomics. 2013 Jul 17;14:481. doi: 10.1186/1471-2164-14-481 (PMC3728262; doi:10.1186/1471-2164-14-481)
Supplement: Additional file 7 — Host range of sequenced phage clusters. PDF file containing the host range of sequenced phages in clusters. [file 1471-2164-14-481-S7.pdf]

Additional file 7. Host range of sequenced phage clusters

	Cluster 1						Cluster 2		Cluster 3		Cluster 4				Cluster 5				Cluster 6	
Salmonella Serovar	SP-019	SP-030	SP-039	SP-088	SP-099	SP-124	SP-029	SP-063	SP-058	SP-076	SP-010	SP-012	SP-107	Felix O1	SP-031	SP-038	SP-049	SP-101	SP-062	SP-069
Typhimurium A4-737	+	+	+	+	-	+	+	+	+	+	-	-	+	+	-	-	-	+	-	-
Typhimurium S53-70	-	+	-	-	-	+	+	+	+	+	-	-	-	+	-	-	-	+	-	-
Enteritidis S5-371	-	-	+	-	-	-	-	+	+	+	-	-	-	+	-	-	-	-	-	-
Copenhagen A4-712	+	+	-	-	-	+	+	+	-	+	-	-	-	+	-	-	-	+	-	-
Newport S5548	+	+	-	+	+	+	+	-	-	-	+	+	-	+	-	-	-	-	+	+
Newport S5-515	+	+	-	-	+	+	+	-	-	+	+	+	-	+	-	-	-	-	+	+
Dublin S5-368	-	+	-	-	-	+	-	+	+	+	-	-	-	+	-	-	-	+	-	-
Dublin R6-535	-	+	-	-	-	+	-	+	+	+	-	-	-	+	-	-	-	+	-	-
Kentucky S5-431	-	-	-	-	-	-	-	-	-	-	-	-	-	+	-	-	-	-	+	-
Kentucky R8-140	-	-	-	-	-	-	-	-	-	-	-	-	-	+	-	-	-	-	+	-
Anatum A4-525	+	-	-	-	-	+	-	-	-	-	-	-	-	+	-	-	-	-	-	-
Mbandaka A4-793	-	+	-	-	-	-	-	-	-	-	+	+	+	+	-	-	-	-	-	-
Agona S5-667	-	-	-	-	-	+	-	-	-	-	-	-	-	+	-	-	-	-	-	-
Agona S9-322	-	-	-	-	-	-	-	-	-	+	-	-	-	+	-	-	-	-	-	-
4,5,12:i:- S5-390	+	+	-	-	-	-	-	+	+	+	-	-	+	+	-	-	-	-	-	-
Montevideo S5-474	-	+	-	-	-	-	-	-	-	-	+	+	-	+	-	-	-	-	-	-
Oranienburg R8-376	-	-	-	-	-	+	-	-	-	-	-	+	-	+	-	-	-	-	-	-
Muenster S5-417	-	-	-	-	-	-	-	-	-	-	-	-	-	+	-	-	-	-	-	-
Heidelberg S5-455	+	-	-	-	-	+	-	+	+	-	-	+	-	+	-	-	-	-	-	-
Infantis S5-506	-	-	-	-	-	-	-	-	-	-	-	-	-	+	-	-	-	-	-	-
Saintpaul S5-369	-	+	-	-	-	+	+	+	+	-	-	-	-	+	-	-	-	-	-	-
Braenderup S5-373	-	-	-	-	-	-	-	-	-	-	-	-	-	+	-	-	-	-	-	-
Cerro R8-242	-	+	+	-	-	+	-	-	-	-	-	+	-	+	+	+	+	-	-	-

+: Lysis was observed, indicating susceptibility

-: No lysis was observed, indicating resistance
